# Retraction: The smoking paradox in ischemic stroke patients treated with intra-arterial thrombolysis in combination with mechanical thrombectomy–VISTA-Endovascular

**DOI:** 10.1371/journal.pone.0279276

**Published:** 2022-12-12

**Authors:** Anna Kufner, Huma Fatima Ali, Martin Ebinger, Jochen B. Fiebach, David S. Liebeskind, Matthias Endres, Bob Siegerink, on behalf of the VISTA-Endovascular Collaborators

After this article [1] was published, the authors became aware of a dataset error that renders the article’s conclusions invalid.

Specifically, due to data labelling and missing information issues, the ‘IAT’ data reflect intra-arterial (IA) treatment rather than the more restricted treatment type of IA-thrombolysis. Further investigation of the dataset revealed that only 24 individuals in the study population received IA-thrombolysis, instead of N = 216 as was reported in [1]. Hence, the article’s main conclusion is not valid or reliable as it is based on the wrong data.

Furthermore, due to the small size of the IA-thrombolysis-positive group, the dataset is not sufficiently powered to address the research question.

In light of the above concerns, the authors retract this article.

All authors agree with retraction.
